# The Relationship Between Social Class and Generalized Trust: The Mediating Role of Sense of Control

**DOI:** 10.3389/fpsyg.2021.729083

**Published:** 2021-09-27

**Authors:** Ruichao Qiang, Xiang Li, Qin Han

**Affiliations:** ^1^Key Laboratory of Intelligent Education Technology and Application of Zhejiang Province, Zhejiang Normal University, Jinhua, China; ^2^Department of Psychology, College of Teacher Education, Zhejiang Normal University, Jinhua, China; ^3^Tin Ka Ping Moral Education Research Center, Zhejiang Normal University, Jinhua, China; ^4^Department of Psychology, School of Educational Science, Shanxi Normal University, Taiyuan, China

**Keywords:** generalized trust, sense of control, social class, socioeconomic status, social trust

## Abstract

The success and well-being theory of trust holds that higher social class is associated with higher generalized trust, and this association has been well documented in empirical research. However, few studies have examined the processes that might explain this link. This study extends this assumption to explore the mediating mechanism in the association. We hypothesized that social class would positively predict generalized trust, and the relationship would be mediated by people’s sense of control. Self-report data were collected from 480 adults (160 males, 320 females; ages 18–61) who participated through an online crowdsourcing platform in China. The results of multiple regression and mediation analyses supported the hypothesized model. This research provides further support for the success and well-being theory of trust, and builds on it by identifying greater sense of control as a possible explanation for the link between high social class and generalized trust. Limitations and possible future research are discussed.

## Introduction

Generalized trust is regarded as the core component of social capital and the building block of modern societies (Fukuyama, 1995; Delhey et al., 2011; Freitag and Bauer, 2013; Kim, 2018). It motivates a range of positive societal outcomes, including economic development (Tabellini, 2010), institutional quality (Robbins, 2012), civic engagement (Delhey et al., 2011) and democracy (Paxton, 2002; Zmerli and Newton, 2008). Without generalized trust, social disorder and conflict are commonplace (Putnam, 2000; Robbins, 2016; Jing, 2019).

One consistent correlate of generalized trust is social class or socio-economic status, with the rich and well-educated reporting more generalized trust than their lower social class counterparts (Putnam, 2000; Alesina and La Ferrara, 2002; Hamamura, 2012; Brandt et al., 2015; Navarro-Carrillo et al., 2018b). The link between social class and generalized trust has been postulated for decades. For instance, Simmel (1950) emphasized that there was the discrepancy of resources that are available to different social class to afford the risks of trust. A few prior studies have investigated potential psychological mechanisms (e.g., relative deprivation) in the social class-interpersonal (dis)trust relationship (Yu et al., 2020). However, there has been a little empirical research regarding the explanation of why social class and generalized trust are correlated. In the current research, we fill this gap by testing the role that sense of control may play in explaining this association.

Generalized trust refers to one’s belief that most people can be trusted (Yamagishi and Yamagishi, 1994; Uslaner, 2002; Freitag and Traunmuller, 2009; Navarro-Carrillo et al., 2018b). People with high generalized trust hold a general belief in human benevolence and they believe that the trustee has benign intentions in social interactions (Yamagishi and Yamagishi, 1994). As a result, they tend to trust strangers, passersby on the street, and other people whom they do not know well. Although generalized trust exposes people to the risk that the target of trust has harmful intentions, this risk may be outweighed by the benefits of trusting strangers. One of the benefits of generalized trust is its promotion on interactions among unfamiliar individuals. Interactions with unfamiliar people expose individuals to novel information and resources that are not available in acquainted relationships (Hamamura, 2012).

Previous research suggested that the risks and benefits of generalized trust are balanced differently across people from different groups, including different social classes (Hamamura, 2012; Brandt et al., 2015). We hold that a sense of personal control may contribute to people’s perception of these risks and benefits of trust. Members of the lower social class are likely to have a lower sense of control, and thus a lower trust to other people. In the following sections we review the literature on the direct relationship between social class and generalized trust, and the literature relevant to our proposal that sense of control may mediate this link.

### Direct Relationship Between Social Class and Generalized Trust

Social class is typically conceptualized as a reflection of multiple features of social life (Fiske and Markus, 2012; Kraus and Keltner, 2013; Daganzo and Bernardo, 2018). Social class is a context rooted in both the resources of social life (e.g., wealth, education, occupation) and the individual’s perceived rank within the social hierarchy (Kraus et al., 2009, 2012). Traditionally, researchers measure social class in terms of objective indicators such as the individual’s level of education, income, and occupation prestige (Kraus et al., 2009, 2012; Daganzo and Bernardo, 2018).

However, there are several inherent problems in assessing social class with objective variables. For instance, it is uncertain how objective indicators (e.g., education, income) combine to yield a composite score representing social class (Kraus et al., 2009; Oakes and Rossi, 2003). As a result, many researchers have questioned the validity of objective metrics of social class in capturing the essence of class. Moreover, many research suggested that subjective measures of social class, compared with the objective measures, more strongly predict the psychological outcomes and serve as a more consistent predictor of social explanation (Adler et al., 2000; Kraus et al., 2011, 2012). Thus, in this study we refer to social class using subjective measures.

According to the success and well-being theory of trust (Delhey and Newton, 2003), generalized trust is more likely to be expressed by people from the upper class than people from the lower class (Alesina and La Ferrara, 2002; Gheorghiu et al., 2009; Hamamura, 2012; Brandt et al., 2015; Navarro-Carrillo et al., 2018b). Trust always carries risks, and it is more risky for lower class individuals (Hamamura, 2012; Navarro-Carrillo et al., 2018b). Lower status individuals who commonly face resource scarcity cannot afford to lose even a little if their trust is betrayed. In contrast, upper class individuals have abundant properties to protect against the risks and vulnerabilities of trust (Brandt et al., 2015), and they can gain more benefits from trust (Delhey and Newton, 2003; Hamamura, 2012).

Moreover, from this perspective, social trust is the product of adult life experiences. Upper class people have been treated with more respect and kindness. Consequently, they are more trusting than lower class individuals who always suffer discrimination and social exclusion (Putnam, 2000). This theory is supported to some degree by survey data provided by the American General Social Survey (Alesina and La Ferrara, 2002) and the German Socio-Economic Panel (Korndörfer et al., 2015). These studies suggest that social class is consistently and positively related to generalized trust.

### Sense of Control as a Potential Mediator

Although previous studies have indicated that social class has enduring association with generalized trust (Hamamura, 2012; Brandt et al., 2015; Navarro-Carrillo et al., 2018b), the specific mechanism involved in this association has been rarely examined. One reason that social class is linked with generalized trust may be that members of different social classes differ in their sense of control. Several studies have documented a disparity in sense of control felt by members of the upper and lower social classes, with upper class individuals typically reporting greater perceived control over their life (Lachman and Weaver, 1998; Kraus et al., 2009; Daganzo and Bernardo, 2018).

Sense of control or self-agency has been described as the experience of being the source of one’ s own actions and their consequences (Dewey et al., 2010; Di Plinio et al., 2020). From an event-control approach (Jordan, 2003), one’s sense of agency depends partly on contextual information about the degree of control an individual has over the environment (Dewey et al., 2010; Kumar and Srinivasan, 2012; Di Plinio et al., 2019). From the social cognition perspective on social class, social class contexts elicit a coherent set of social cognitive patterns of thought, feeling, and action with regard to oneself and other people (Kraus et al., 2012). Specially, people from upper class inhabit an environment with abundant resources, personal freedom, and social opportunities. This makes them perceive a greater sense of personal control over life (Kraus et al., 2009, 2012). Furthermore, upper class individuals are more likely to occupy positions of influence and elevated status, which strongly promote their perceived personal control (Kraus et al., 2012). In contrast, the social contexts of lower class are characterized by reduced resources, external threats, and vulnerability, which may make them feel powerless to exert control over their lives (Haushofer and Fehr, 2014; Piff, 2014).

Furtherly, the social class difference in sense of control may lead to the disparity in generalized trust (Navarro-Carrillo et al., 2018a; Samson and Zaleskiewicz, 2020). Uslaner (2002), for instance, views individuals’ sense of control over their life as key to understanding their trust in people. Generalized trust always carries risks due to the possible betrayal by others (Delhey and Newton, 2003; Hamamura, 2012; Navarro-Carrillo et al., 2018b). Individuals with greater sense of control can afford to maintain an optimistic view of other people and be more trusting in general (Samson and Zaleskiewicz, 2020). In contrast, individuals with lower perceived personal control over life are psychologically defensive and prefer to distrust others (Brandt and Henry, 2012; Samson and Zaleskiewicz, 2020). It makes sense to think that people lack of perceived control express diminished generalized trust.

## Current Study

The main purpose of the present research was to examine the relationships among social class, generalized trust and sense of control. We expected to find further evidence of the social class difference in generalized trust, as it has been documented in many other studies. More importantly, we tested a model in which sense of control mediated the relationship between social class and generalized trust. The four hypotheses below were derived from the theoretical assumptions and empirical evidence presented above.

H1 Social class will significantly and positively predict generalized trust.

H2 Social class will be positively related to sense of control.

H3 Sense of control will be positively associated with generalized trust.

H4 Social class will be associated with higher generalized trust through heightened sense of control.

## Materials and Methods

### Participants

An online crowdsourcing platform in mainland China, which provides functions equivalent to Amazon Mechanical Turk, recruited 494 Chinese participants. Of these, 14 reported their age to be below 18. These participants were excluded from the following analyses, leaving a final sample of 480 individuals (160 males, 320 females). The age of participants ranged from 18 to 61 years of age (*M* = 27.77, *SD* = 8.21).

A sensitivity power analysis using G*Power (Faul et al., 2007) indicated that, the minimum effect size required to produce power at the 0.80 level in linear multiple regression with current sample size was 0.016. The effect size of regression coefficients in our study were all greater than it.

### Procedure

Participants were instructed that they would participate in an online survey about their social attitudes. They were informed that their answers would be anonymous and that they could stop participating at any time. They signed an informed consent form prior to participating in the online surveys. Then, they filled out measures of social class, sense of control, and generalized trust. The participants also provided their gender and age. It took about four min to complete all the scales. If participants skipped an item, they were reminded to complete it when they clicked the submit button. The survey could not be submitted until all items were completed. This provided a data set with no missing values. The participants were thanked for participating in the study but received no other reward.

### Measures

#### Social Class

We assessed social class using the MacArthur Ladder Scale (Adler et al., 2000). Participants were shown a picture of a 10-rung ladder and asked to imagine that the ladder represented where people stand in society. They were told that at the bottom (social class = 0) are the people who are the worst off—who have the least education, the least money, and the least respected jobs or no jobs; at the top of the ladder (social class = 10) are the people who are the best off—those who have the most education, the most money, and the most respected jobs. Then, they were asked to indicate their position at the ladder at this time of their life relative to other people in society (*M* = 4.55, *SD* = 1.69).

#### Sense of Control

Sense of control was assessed using the established measure from Lachman and Weaver (1998). The Sense of Control Scale is composed of 12 items—4 measuring personal mastery and 8 measuring perceived constraints. Sample items are: “*I can do just about anything that I really set my mind to*” and “*When I really want to do something, I usually find a way to succeed at it*” from the personal mastery dimension, and “*Other people determine most of what I can and cannot do*” and “*There is little I can do to change many of the important things in my life*” from the perceived constraints dimension. Items were rated on a 7-point Likert scale (1 = *strongly disagree*, 7 = *strongly agree*). The items belonging to perceived constraints dimension were reverse-scored, then all items were averaged to obtain a composite score for sense of control (α = 0.82). Confirmatory Factor Analysis showed that the scale had high construct validity in this study (*CFI* = 0.95, *TLI* = 0.94, *RMSEA* = 0.06, 90% *CI* [0.04, 0.07], *SRMR* = 0.05).

#### Generalized Trust

Generalized trust was assessed using an established three-item measure (Chen et al., 2011). To assess generalized trust, participants indicated their agreement with three statements. The first item is the classic binary trust question from the World Value Survey: “*Generally speaking, would you say that most people can be trusted or that you need to be very careful in dealing with people?*” Responses were coded as 1 = *need to be very careful*, 2 = *don’t know*, 3 = *most people can be trusted.* The second item is “*Do you think that most people will take advantage of your weakness or that they will do you justice?*” with responses coded as 1 = *take advantage of me*, 2 = *a 50–50 chance*, 3 = *do me justice*. The third item is “*No matter known or not, most people are trustworthy.*” Responses were coded as 1 = *they aren’t trustworthy*, 2 = *a 50–50 chance*, 3 = *they are trustworthy*. Scores on the three items were averaged to form the generalized trust scale. The Cronbach’s alpha coefficient in this study was 0.62.

## Results

Prior to the main analyses, we conducted a preliminary analysis among variables.^1^ Correlations between primary variables of interest and demographic variables were all not significant. However, prior research has controlled for gender and age when analyzing the contribution of social class to social trust (Hamamura, 2012; Brandt et al., 2015; Kim et al., 2021). Therefore, and as they are common sociodemographic variables, we consider their effects on the hypothesized associations.

Then, we performed regression analyses predicting the links among social class, sense of control and generalized trust. We included gender and age as control variables to determine whether the associations held beyond the effects of demographic variables. The results of these analyses are summarized in Table 1.

**TABLE 1:** Relationships among social class, sense of control, and generalized trust.

	Class-trust relationship	Class-control relationship	Control-trust relationship
**Without control variables**	β = 0.23, *F*(1, 478) = 26.10	β = 0.33, *F*(1, 478) = 56.31	β = 0.30, *F*(1, 478) = 47.05
**With control variables**	β = 0.23, *F*(2, 476) = 8.91	β = 0.33, *F*(2, 476) = 20.47	β = 0.30, *F*(2, 476) = 15.66

*Note: All effects are significant at p < 0.001. The control variables were gender and age.*

Supporting H1, social class was significantly associated with generalized trust, *R*^2^ = 0.052, *F*(1, 478) = 26.10, β = 0.23, *p* < 0.001, 95% *CI* [0.140, 0.315]. This relationship remained significant when controlling for gender and age, *R*^2^ = 0.053, *F*(2, 476) = 8.91, β = 0.23, *p* < 0.001, 95% *CI* [0.140, 0.316]. Supporting H2, social class predicted greater sense of control, *R*^2^ = 0.105, *F*(1, 478) = 56.31, β = 0.33, *p* < 0.001, 95% CI [0.240, 0.410]. This link remained significant when controlling age and gender, *R*^2^ = 0.114, *F*(2, 476) = 20.47, β = 0.33, *p* < 0.001, 95% CI [0.241, 0.410]. H3 was also confirmed. Sense of control predicted significantly greater generalized trust, *R*^2^ = 0.09, *F*(1, 478) = 47.05, β = 0.30, *p* < 0.001, 95% CI [0.214, 0.385]. This result was still significant after controlling for gender and age, *R*^2^ = 0.09, *F*(2, 476) = 15.66, β = 0.30, *p* < 0.001, 95% CI [0.213, 0.385].

**FIGURE 1:**
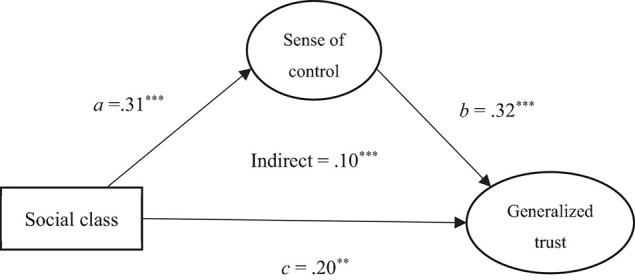
Mediating model illustrating the relationships among social class, sense of control, and generalized trust. Paths a, b, and c represent direct effects. All numbers are standardized regression coefficients. **p < 0.01, ***p < 0.001. The 95% confidence interval of the bias-corrected indicates a significant indirect effect.

To determine whether sense of control acted as a mediator between social class and generalized trust, we tested the hypothesized mediation model in Amos 27. Structural equation modeling indicated that the hypothesized model (Figure 1) showed a good fit with the data (*CFI* = 0.86, *TLI* = 0.83, *RMSEA* = 0.07, 90% *CI* [0.06, 0.08]). We used a bootstrapping technique with 5,000 iterations to estimate the indirect effect of social class on generalized trust through perceived control. The size of the indirect effect was estimated by examining the 95% bootstrap confidence interval (*CI*) of the estimate; the effect is considered significant when the *CI* does not include zero. Supporting H4, the indirect effect was significant; that is, higher social class was associated with higher generalized trust via a process of greater sense of control (β = 0.10, *SE* = 0.03, *p* < 0.001, bias-corrected 95% *CI* [0.05, 0.18]). As can be seen in Figure 1, the direct effect of social class on generalized trust remained significant (β = 0.20, *SE* = 0.06, *p* = 0.003, bias-corrected 95% *CI* [0.07, 0.31]) after including the mediation component, suggesting partial mediation.

## Discussion

It is well documented that members of the upper social class show more generalized trust than members of the lower social class, but a little research has examined the reason for this association. The present study tested whether sense of personal control plays a mediating role in the association between social class and social trust. We found evidence that supported four key hypotheses derived from the success and well-being theory of trust (Delhey and Newton, 2003). This study represents the first empirical demonstration of a mediator of sense of control between social class and generalized trust, and the new evidence that this is a process through which social cognition effect of social class (Kraus et al., 2012) can operate.

This study enriches the growing body of research on social class and trust. As expected, social class significantly and positively predicted generalized trust. This finding is consistent with the success and well-being theory of trust that asserts a positive association between social class and generalized trust (Delhey and Newton, 2003; Brandt et al., 2015; Edelman, 2017). The present study supports this long-held view and adds new evidence that helps explain why higher social class is associated with greater generalized trust.

The results showed that perceived control may be a mediating psychological mechanism in the association between social class and trust beliefs. As the social cognition perspective on social class suggests (Kraus et al., 2012), social class contexts elicit a coherent set of social cognitive patterns of thought, including the perception of personal control. Specifically, the upper social class context generates a stronger sense of control than the lower social class context (Kraus et al., 2009; Daganzo and Bernardo, 2018). This is consistent with the event-control approach, which asserts that context information can modulate individual’s sense of control (Jordan, 2003; Di Plinio et al., 2019). Furthermore, several researchers have highlighted generalized trust as a direct consequence of sense of personal control (Uslaner, 2002; Navarro-Carrillo et al., 2018a). Samson and Zaleskiewicz (2020) declared that people who have a strong sense of control over one’s own life may be more likely to maintain an optimistic view of other people and to be more trusting in general. Sense of control thus is a psychological mechanism that links social class to trust and a helpful focus of intervention for people of lower class who struggle with trusting others.

The current study not only supports the success and well-being theory of trust, but extends the theory by revealing that the cognitive factors work when social class may serve to structure social psychological functioning. Furthermore, a new question is raised and might need to be incorporated into the success and well-being theory of trust. That is, whether emotional factors act in the function process of social class, given that some negative emotion such as insecurity and anxiety are the powerful attenuators of trust (Patterson, 1999; Nguyen, 2017; Navarro-Carrillo et al., 2018a).

The present work is not without limitations. First, the cross-sectional design limits the causal conclusions that can be drawn from the data. Given that social class involves a long-term experience and is probably more stable than generalized trust, it may be that social class influences generalized trust in the association. However, it is also possible that a third variable affects both of them. For example, social class of parents may partially determine the social class of children and influence the trust belief of children through the socialization of social cognition in the family.

Secondly, the internal reliability of the generalized trust scale was acceptable but not high. This is a common weakness of short scales (Tavakol and Dennick, 2011). Cronbach’s alpha, a commonly used measure of internal consistency, is affected by the length of the scale. If the scale length is too short, the value of alpha is reduced (Streiner, 2003; Tavakol and Dennick, 2011). However, this measure effectively exhibited social class tendency of generalized trust in our sample, and a similar measure has been used in other research on social class and generalized trust (Brandt et al., 2015). Nevertheless, a longer scale may be more useful in future research, such as the General Trust Scale (Yamagishi and Yamagishi, 1994) which has 6 items and showed high internal consistency (alpha values range from 0.71 to 0.74) in other studies (Navarro-Carrillo et al., 2018a,b).

Thirdly, we used only subjective measures to assess social class, and different results may be obtained using objective measures. Subjective measures have been found to be more potent predictors of psychological outcomes than the objective measures (Adler et al., 2000; Kraus et al., 2011, 2012). However, in some cases, objective measures of social class work better in predicting social explanations (Kim et al., 2021). As a consequence, including both subjective and objective measures of social class would allow a test of which aspects of social class are most predictive of generalized trust. It might also help in inspecting the inter-relations between objective vs. subjective social class.

Fourthly, the role of psychological defensiveness playing in the association between social class and generalized trust should be further explored. People from the lower class face long-term prejudice and psychological threats to the self, which make them psychologically defensive against these self-threats (Henry, 2009; Brandt et al., 2015). A manifestation of psychological defensiveness is in terms of distrust in other people (Brandt and Henry, 2012). Psychological defensiveness may be an individual difference that would explain some of the variability in generalized trust among people of the lower social class.

Lastly, cultural issues should be considered in interpreting the results. Culture exists as a socially shared reality that generates values, beliefs, and social interaction norms in social life (Barker, 2017). For instance, some cultures value interdependence and benevolence while others value independence and competitiveness. Consequently, social trust is generally affected by cultural elements (Gheorghiu et al., 2009; Berigan and Irwin, 2011; Steel et al., 2018). This may lead to diverse baselines of social trust across different cultures and, in turn, may impact the association between social class and trust. This effect may function through some general cultural factors (e.g., individualism vs. collectivism, multiculturalism, politics regarding immigration) or through attention to emotional and cognitive stimuli (Grossmann et al., 2012).

## Conclusion

There is a well-documented link between social class and generalized trust (Hamamura, 2012; Brandt et al., 2015). However, a little research has examined the reason for this link. The key contribution of the present study is the finding that sense of control acts as a mediator between social class and generalized trust. Members of the upper social class were inclined to perceive high control over their outcomes, and they held a strong generalized trust in daily life. In contrast, members of the lower social class were more likely to feel a low sense of control, and in turn, low social trust.

## Data Availability Statement

The raw data supporting the conclusions of this article will be made available by the authors, without undue reservation.

## Ethics Statement

The study involving human participants were reviewed and approved by the Department of psychology of the Zhejiang Normal University. Written informed consent to participate in the study was provided by the participants or where applicable, the participants legal guardian/next of kin. The patients/participants provided their written informed consent to participate in this study. Written informed consent was obtained from the individual (s) for the publication of any potentially identifiable images or data included in this article.

## Author Contributions

RQ conceived the study, analyzed relevant literature, and wrote the manuscript in all its sections. XL collected the data, conducted the statistical analysis of the data, wrote the current version of the results section. QH conceived the study, analyzed relevant literature, structured the questionnaire, and wrote the results and conclusion sections. All authors contributed to the article and approved the submitted version.

## Conflict of Interest

The authors declare that the research was conducted in the absence of any commercial or financial relationships that could be construed as a potential conflict of interest.

## Publisher’s Note

All claims expressed in this article are solely those of the authors and do not necessarily represent those of their affiliated organizations, or those of the publisher, the editors and the reviewers. Any product that may be evaluated in this article, or claim that may be made by its manufacturer, is not guaranteed or endorsed by the publisher.

## Footnotes

1 In our analyses, gender was coded as a dummy variable, male = 1, female = 2. All the variables were standardized before the analyses.

## References

[B1] Adler N. E. Epel E. S. Castellazzo G. Ickovics J. R. (2000). Relationship of subjective and objective social status with psychological and physiological functioning: preliminary data in healthy white women. *Health Psychol.* 19 586–592. 10.1037/0278-6133.19.6.586 11129362

[B2] Alesina A. La Ferrara E. (2002). Who trusts others? *J. Public Econ.* 85 207–234. 10.1016/S0047-2727(01)00084-6

[B3] Barker G. G. (2017). Acculturation and bicultural integration in organizations: conditions, contexts, and challenges. *Int. J. Cross Cult. Manag.* 17 281–304. 10.1177/1470595817712741

[B4] Berigan N. Irwin K. (2011). Culture, cooperation, and the general welfare. *Soc. Psychol. Q.* 74 341–360. 10.1177/0190272511422451

[B5] Brandt M. J. Henry P. J. (2012). Psychological defensiveness as a mechanism explaining the relationship between low socioeconomic status and religiosity. *Int. J. Psychol. Relig.* 22 321–332. 10.1080/10508619.2011.646565

[B6] Brandt M. J. Wetherell G. Henry P. J. (2015). Changes in income predict change in social trust: a longitudinal analysis. *Polit. Psychol.* 36 761–768. 10.1111/pops.12228

[B7] Chen J. Huhe N. Lu C. (2011). Causal mechanisms between social trust and community governance. *Chin. J. Sociol.* 31 22–40. 10.15992/j.cnki.31-1123/c.2011.06.003

[B8] Daganzo M. A. A. Bernardo A. B. (2018). Socioeconomic status and problem attributions: the mediating role of sense of control. *Cogent Psychol.* 5:1525149. 10.1080/23311908.2018.1525149

[B9] Delhey J. Newton K. (2003). Who trusts? The origins of social trust in seven nations. *Eur. Soc.* 5 93–137. 10.1080/1461669032000072256

[B10] Delhey J. Newton K. Welzel C. (2011). How general is trust in ‘most people’? Solving the radius of trust problem. *Am. Sociol. Rev.* 76 786–807. 10.1177/0003122411420817

[B11] Dewey J. A. Seiffert A. E. Carr T. H. (2010). Taking credit for success: the phenomenology of control in a goal directed task. *Conscious. Cogn.* 19 48–62. 10.1016/j.concog.2009.09.007 19833535

[B12] Di Plinio S. Arno S. Perrucci M. G. Ebisch S. J. (2019). Environmental control and psychosis-relevant traits modulate the prospective sense of agency in non-clinical individuals. *Conscious. Cogn.* 73:102776. 10.1016/j.concog.2019.102776 31272013

[B13] Di Plinio S. Perrucci M. G. Aleman A. Ebisch S. J. (2020). I am Me: brain systems integrate and segregate to establish a multidimensional sense of self. *Neuroimage* 205:116284. 10.1016/j.neuroimage.2019.116284 31629830

[B14] Edelman (2017). *2017 Edelman Trust Barometer-Global report.* Available online at: https://www.slideshare.net/EdelmanInsights/2017-edelman-trust-barometer-global-results-71035413 (accessed March 27, 2019.

[B15] Faul F. Erdfelder E. Lang A.-G. Buchner A. (2007). G*Power 3: a flexible statistical power analysis program for the social, behavioral, and biomedical sciences. *Behav. Res. Methods* 39 175–191. 10.3758/BF03193146 17695343

[B16] Fiske S. T. Markus H. R. (eds) (2012). *Facing Social Class: How Societal Rank Influences Interaction.* New York, NY: Russell Sage Foundation.

[B17] Freitag M. Bauer P. C. (2013). Testing for measurement equivalence in surveys. *Public Opin. Q.* 77 24–44. 10.1093/poq/nfs064

[B18] Freitag M. Traunmuller R. (2009). Spheres of trust: an empirical analysis of the foundations of particularised and generalised trust. *Eur. J. Polit. Res.* 48 782–803. 10.1111/j.1475-6765.2009.00849.x

[B19] Fukuyama F. (1995). *Trust: The Social Virtues and the Creation of Prosperity.* New York, NY: Free Press.

[B20] Gheorghiu M. Vignoles V. Smith P. (2009). Beyond the United States and Japan: testing Yamagishi’s emancipation theory of trust across 31 nations. *Soc. Psychol. Q.* 72 365–383. 10.1177/019027250907200408

[B21] Grossmann I. Ellsworth P. C. Hong Y. Y. (2012). Culture, attention, and emotion. *J. Exp. Psychol. Gen.* 141 31–36. 10.1037/a0023817 21639670

[B22] Hamamura T. (2012). Social class predicts generalized trust but only in wealthy societies. *J. Cross Cult. Psychol.* 43 498–509. 10.1177/0022022111399649

[B23] Haushofer J. Fehr E. (2014). On the psychology of poverty. *Science* 344 862–867. 10.1126/science.1232491 24855262

[B24] Henry P. J. (2009). Low-status compensation: a theory for understanding the role of status in cultures of honor. *J. Pers. Soc. Psychol.* 97 451–466. 10.1037/a0015476 19686001

[B25] Jing S. (2019). “Conceptualizing and measuring sense of social trust,” in *Social Mentality in Contemporary China*, ed. Yang Y. (Singapore: Springer), 87–109. 10.1007/978-981-13-7812-6_7

[B26] Jordan J. S. (2003). Emergence of self and other in perception and action: an event-control approach. *Conscious. Cogn.* 12 633–646. 10.1016/S1053-8100(03)00075-8 14656506

[B27] Kim H. S. (2018). Particularized trust, generalized trust, and immigrant self-rated health: cross-national analysis of World Values Survey. *Public Health* 158 93–101. 10.1016/j.puhe.2018.01.039 29588067

[B28] Kim Y. Sommet N. Na J. Spini D. (2021). Social class—not income inequality—predicts social and institutional trust. *Soc. Psychol. Personal. Sci.* 10.1177/1948550621999272 [Epub ahead of print].

[B29] Korndörfer M. Egloff B. Schmukle S. C. (2015). A large scale test of the effect of social class on prosocial behavior. *PLoS One* 10:e0133193. 10.1371/journal.pone.0133193 26193099 PMC4507988

[B30] Kraus M. W. Horberg E. J. Goetz J. L. Keltner D. (2011). Social class rank, threat vigilance, and hostile reactivity. *Pers. Soc. Psychol. Bull.* 37 1376–1388. 10.1177/0146167211410987 21653579

[B31] Kraus M. W. Keltner D. (2013). Social class rank, essentialism, and punitive judgment. *J. Pers. Soc. Psychol.* 105 247–261. 10.1037/a0032895 23713698

[B32] Kraus M. W. Piff P. K. Keltner D. (2009). Social class, sense of control, and social explanation. *J. Pers. Soc. Psychol.* 97 992–1004. 10.1037/a0016357 19968415

[B33] Kraus M. W. Piff P. K. Mendoza-Denton R. Rheinschmidt M. L. Keltner D. (2012). Social class, solipsism, and contextualism: how the rich are different from the poor. *Psychol. Rev.* 119 546–572. 10.1037/a0028756 22775498

[B34] Kumar D. Srinivasan N. (2012). Hierarchical event-control and subjective experience of agency. *Front. Psychol.* 3:410. 10.3389/fpsyg.2012.00410 23181026 PMC3501285

[B35] Lachman M. E. Weaver S. L. (1998). The sense of control as a moderator of social class differences in health and well-being. *J. Pers. Soc. Psychol.* 74 763–773. 10.1037/0022-3514.74.3.763 9523418

[B36] Navarro-Carrillo G. Valor-Segura I. Lozano L. M. Moya M. (2018a). Do economic crises always undermine trust in others? The case of generalized, interpersonal, and in-group trust. *Front. Psychol.* 9:1955. 10.3389/fpsyg.2018.01955 30374321 PMC6196242

[B37] Navarro-Carrillo G. Valor-Segura I. Moya M. (2018b). Do you trust strangers, close acquaintances, and members of your ingroup? Differences in trust based on social class in Spain. *Soc. Indic. Res.* 135 585–597. 10.1007/s11205-016-1527-7

[B38] Nguyen C. (2017). Labour market insecurity and generalized trust in welfare state context. *Eur. Sociol. Rev.* 33 225–239. 10.1093/esr/jcw058

[B39] Oakes J. M. Rossi R. H. (2003). The measurement of SES in health research: current practice and steps toward a new approach. *Soc. Sci. Med.* 56 769–784. 10.1016/S0277-9536(02)00073-4 12560010

[B40] Patterson O. (1999). “Liberty against the democratic state: on the historical and contemporary sources of American distrust,” in *Democracy and Trust*, ed. Warren M. E. (Cambridge: Cambridge University Press), 151–207. 10.1017/CBO9780511659959.006

[B41] Paxton P. (2002). Social capital and democracy: an independent relationship. *Am. Sociol. Rev.* 67 254–277. 10.2307/3088895

[B42] Piff P. K. (2014). Wealth and the inflated self: class, entitlement, and narcissism. *Pers. Soc. Psychol. Bull.* 40 34–43. 10.1177/0146167213501699 23963971

[B43] Putnam R. D. (2000). *Bowling Alone: The Collapse and Revival of American community.* New York, NY: Simon and Schuster.

[B44] Robbins B. G. (2012). Institutional quality and generalized trust: a nonrecursive causal model. *Soc. Indic. Res.* 107 235–258. 10.1007/s11205-011-9838-1

[B45] Robbins B. G. (2016). From the general to the specific: how social trust motivates relational trust. *Soc. Sci. Res.* 55 16–30. 10.1016/j.ssresearch.2015.09.004 26680285

[B46] Samson K. Zaleskiewicz T. (2020). Social class and interpersonal trust: Partner’s warmth, external threats and interpretations of trust betrayal. *Eur. J. Soc. Psychol.* 50 634–645. 10.1002/EJSP.2648

[B47] Simmel G. (1950). *The Sociology of Georg Simmel.* Glencoe, IL: Free Press.

[B48] Steel P. Taras V. Uggerslev K. Bosco F. (2018). The happy culture: a theoretical, meta-analytic, and empirical review of the relationship between culture and wealth and subjective well-being. *Pers. Soc. Psychol. Rev.* 22 128–169. 10.1177/1088868317721372 28770649 PMC5892848

[B49] Streiner D. (2003). Starting at the beginning: an introduction to coefficient alpha and internal consistency. *J. Pers. Assess.* 80 99–103. 10.1207/S15327752JPA8001_18 12584072

[B50] Tabellini G. (2010). Culture and institutions: economic development in the regions of Europe. *J. Eur. Econ. Assoc.* 8 677–716. 10.1111/j.1542-4774.2010.tb00537.x

[B51] Tavakol M. Dennick R. (2011). Making sense of Cronbach’s alpha. *Int. J. Med. Educ.* 2 53–55. 10.5116/ijme.4dfb.8dfd 28029643 PMC4205511

[B52] Uslaner E. M. (2002). *The Moral Foundations of Trust.* Cambridge: Cambridge University Press.

[B53] Yamagishi T. Yamagishi M. (1994). Trust and commitment in the United States and Japan. *Motiv. Emot.* 18 129–166. 10.1007/BF02249397

[B54] Yu G. Zhao F. Wang H. Li S. (2020). Subjective social class and distrust among Chinese college students: the mediating roles of relative deprivation and belief in a just world. *Curr. Psychol.* 39 2221–2230. 10.1007/s12144-018-9908-5

[B55] Zmerli S. Newton K. (2008). Social trust and attitudes toward democracy. *Public Opin. Q.* 72 706–724. 10.1093/poq/nfn054

